# Assessing Lymphatic Uptake of Lipids Using Magnetic Resonance Imaging: A Feasibility Study in Healthy Human Volunteers with Potential Application for Tracking Lymph Node Delivery of Drugs and Formulation Excipients

**DOI:** 10.3390/pharmaceutics13091343

**Published:** 2021-08-27

**Authors:** Adelaide Jewell, Hannah Williams, Caroline L. Hoad, Paul R. Gellert, Marianne B. Ashford, James Butler, Snow Stolnik, David Scurr, Michael J. Stocks, Luca Marciani, Penny A. Gowland, Pavel Gershkovich

**Affiliations:** 1 School of Pharmacy, University of Nottingham, Nottingham NG7 2QL, UK; paxaj3@nottingham.ac.uk (A.J.); Pazss@exmail.nottingham.ac.uk (S.S.); pazdjs@exmail.nottingham.ac.uk (D.S.); pazmjs@exmail.nottingham.ac.uk (M.J.S.); 2 Sir Peter Mansfield Imaging Centre, School of Physics and Astronomy, University of Nottingham, Nottingham NG7 2QX, UK; mszhgw@exmail.nottingham.ac.uk (H.W.); ppzclh@exmail.nottingham.ac.uk (C.L.H.); Ppzpag@exmail.nottingham.ac.uk (P.A.G.); 3 National Institute for Health Research (NIHR), Nottingham Biomedical Research Centre, Nottingham University Hospitals NHS Trust and University of Nottingham, Nottingham NG7 2UH, UK; mszlm1@exmail.nottingham.ac.uk; 4 Advanced Drug Delivery Pharmaceutical Sciences, R&D, AstraZeneca, Macclesfield SK10 2NA, UK; paul.gellert@astrazeneca.com (P.R.G.); marianne.ashford@astrazeneca.com (M.B.A.); 5 GlaxoSmithKline Research and Development, Ware, Hertfordshire SG12 0DP, UK; james.m.butler@gsk.com; 6 Nottingham Digestive Diseases Centre, School of Medicine, University of Nottingham, Nottingham NG7 2UH, UK

**Keywords:** intestinal lymphatic transport, lymph nodes, MRI, lipids, lipid-based formulations

## Abstract

Dietary lipids and some pharmaceutical lipid excipients can facilitate the targeted delivery of drugs to the intestinal lymphatics. Here, the feasibility of magnetic resonance imaging (MRI) for imaging lipid uptake into the intestinal lymphatics was assessed, shedding light on which lymph nodes can be targeted using this approach. Three healthy male volunteers were scanned at 3.0 T at baseline, 120, 180, 240, and 300 min post high-fat meal. A sagittal multi-slice image was acquired using a diffusion-weighted whole-body imaging sequence with background suppression (DWIBS) (pre inversion TI = 260 ms). Changes in area, major, and minor axis length were compared at each time point. Apparent diffusion coefficient (ADC) was calculated (b = 0 and 600 s/mm^2^) across eight slices. An average of 22 nodes could be visualised across all time points. ADC increased at 120 and 180 min compared to the baseline in all three participants by an average of 9.2% and 6.8%, respectively. In two participants, mean node area and major axis lengths increased at 120 and 180 min relative to the baseline. In conclusion, the method described shows potential for repeated lymph node measurements and the tracking of lipid uptake into the lymphatics. Further studies should focus on methodology optimisation in a larger cohort.

## 1. Introduction

The intestinal lymphatic system is responsible for the absorption of dietary lipids from the small intestine in the form of large lipoproteins assembled in the enterocytes (chylomicrons). Drugs with specific physiochemical properties including LogD_7.4_ > 5 have been shown to associate with chylomicrons. This means that they could avoid hepatic first-pass metabolism though delivery to mesenteric lymph nodes, thus bypassing the liver [1]. The use of lipid-based formulations to promote the formation of chylomicrons can further enhance lymphatic targeting. Using this delivery approach, it was shown in rats that up to 250-fold times higher cannabinoid concentrations could be achieved in lymph relative to plasma [2]. Furthermore, lymphatic uptake of drugs with poor chylomicron association can be enhanced through the design of highly lipophilic prodrugs, as recently demonstrated [3,4,5]. Although rats have fewer lymph nodes compared to humans, the basic morphologic structure and rates of lipid transport to the mesenteric nodes are comparable [6,7]. Based on this, it can be assumed that the lymph nodes of human mesentery may also be targeted through the use of chylomicron-associated drugs. In addition, efferent lymph from the mesenteric lymph nodes is understood to collect into the retroperitoneal lymph nodes before entry into the cistern chyli and thoracic duct [8]. Retroperitoneal nodes may also be exposed to chylomicron-associated drugs. However, to our knowledge, very few attempts have been made to quantify drug delivery to the intestinal lymphatic system following oral delivery in humans [9]. With a growing interest surrounding lymphatic targeting using lipid based formulations in the literature [10,11,12], knowledge of which specific nodes can be targeted is essential for determining the clinical potential of intestinal lymphatic drug targeting.

The intestinal lymph nodes are fundamental for response to external pathogens in the diet [13]. They also play important homeostatic roles in intestinal interstitial fluid balance and the uptake of dietary lipids. Unfortunately, the intestinal lymphatic system is also central to a number of pathologies including inflammatory and autoimmune diseases, infectious diseases, lymphomas, and cancer metastasis [12]. Cancer metastasis is widely understood to be responsible for around 90% of cancer-related morbidities [14] and lymphatic involvement is commonly detectable at the time of primary tumour diagnosis. The primary treatment for lymph node metastasis is lymphadenectomy surgery, in which the affected lymph nodes are excised, usually followed by adjuvant chemotherapy or radiotherapy. However, complexity surrounding the identification of sentinel nodes makes this difficult. Subsequently, reoccurrence or unnecessary disruption in lymphatic flow resulting in pain and oedema can occur [15]. In addition, the cytotoxic nature of most clinically relevant chemotherapeutic drugs results in a range of associated adverse effects. Moreover, permeability into affected lymph nodes is often poor [4,16]. Therefore, drugs that specifically target metastatic lymph nodes of the intestines have the potential for significant clinical impact.

The number of metastatic nodes and distance from the primary tumour are major clinical considerations in staging and subsequent prognosis [17]. In many cancers, the pattern of lymph node metastasis from the primary tumours is well described and predictable. Unsurprisingly, the first nodes in which oncological changes can be identified, termed the sentinel node or nodes, are almost always those in closest proximity to the primary tumour. Based on this, metastatic mesenteric lymph nodes are commonly described in gastric, small intestine endocrine, and colorectal cancers [18]. More specifically, colonic carcinoma is the third leading cause of cancer related death in the world [19] and MLN involvement was described in 40% of patients with late stage colorectal cancer [20]. However, in addition to GI tract related cancers, metastasis from primary tumours such as carcinomas in the ovaries, oesophagus, appendix, breast, lung, pancreas, bladder, melanoma as well as Kaposi sarcoma following HIV infection has also been described in the mesenteric lymph nodes [18]. This highlights the incomplete understanding of metastatic processes from these areas. Knowledge to which lymph nodes the drug is delivered could prove to be invaluable for informing which primary tumour metastases could be treated by intestinal lymphatic targeting approach.

Imaging of the intestinal lymph nodes poses a number of difficulties including motion due to peristalsis and respiration, their small size, and central location inside the body. Imaging in the transverse plane has the benefit of allowing the nodes throughout the mesentery that fan out to the left and right of the body to be imaged [1,15,18]. However, the majority of superior mesenteric lymph nodes seen at imaging lie at the root of the superior mesenteric artery (SMA) [21]. The SMA branches from the aorta lying along the spine at the first lumbar vertebra [22]. Therefore imaging in the sagittal plane allows for visualisation of these nodes as well as those closely associated with the aorta. In addition, in house data suggest that abdominal scanning in the sagittal plane reduces motion due to respiration, resulting in a better quality image. Magnetic resonance imaging (MRI) in the sagittal plane was recently described for the imaging of the intestinal lymph nodes in healthy human volunteers [23]. In this work, several parameters were measured including lymph node major and minor axis length and apparent diffusion coefficient (ADC), a measure of the extent of water diffusion. Large lipid droplets have been observed in mesenteric lymph nodes as a result of a high fat diet [24]. Based on this, it was hypothesised that lymph nodes may increase in size as they receive lymph containing dietary lipids. In addition, as the composition of lymph draining into lymph nodes becomes more lipid rich, it was also hypothesised that the ADC may also change. Using these parameters as indications of lipid uptake, the MRI method previously described, may thus allow for the identification of nodes involved in lymphatic uptake of dietary lipids, lipid excipients, and co-administered drugs.

In this work, we aimed to assess the feasibility of MRI for the repeated imaging of the intestinal lymph nodes pre- and post-high fat meal. By analysing these lymph nodes, we also aimed to access the potential of MRI methodology for imaging the distribution and movement of lipids in the intestinal lymphatics. We discuss the relevance of this work in identifying which lymph nodes may be targeted through intestinal lymphatic transport of orally administered drugs and thus the clinical potential of this targeting approach.

## 2. Materials and Methods

The study protocol was approved by the University of Nottingham School of Medicine and Health Sciences Research Ethics Committee, Queens Medical Centre, Nottingham, University Hospitals, Nottingham, UK. (ref no. 358-1906). All subjects gave written informed consent and had no contraindications to MRI.

### 2.1. Study Participants

All three participants were healthy male volunteers aged 25–30 years with no history of underlying cardiac or gastro-intestinal disorders or symptoms. All participants had a healthy BMI (between 18.5 and 24.9) and no food intolerances or allergies.

### 2.2. Study Design

Participants were asked to fast from 10 pm the evening prior to the MRI study day. Water was allowed up until 2 h prior to scanning. Participants were scanned in the supine position at fasted baseline and then 120, 180, 240, and 300 min after the consumption of a high fat content meal. The meal consisted of 300 g creamed rice pudding uniformly mixed with 25 g seedless raspberry jam and 30 g double cream, and a drink of 100 mL orange juice with 240 mL of water (total energy content 518.8 kcal, fat content 18.5 g, carbohydrate content 76.5 g, with a median gastric half-emptying time of 80 min [25]).

### 2.3. MRI Acquisition

All images were acquired using a Philips 3T Ingenia (Best, The Netherlands) with a 32 channel dStream torso coil (Philips Healthcare).

### 2.4. Lymph Node Imaging

The diffusion-weighted whole-body imaging with background suppression (DWIBS) sequence was used to highlight the lymph nodes within the abdomen (pre inversion, TI = 260 ms, for background suppression). A free breathing coronal DWIBS was first acquired to gain a general idea of where nodes were located. Following this, a respiratory triggered DWIBS was performed in the sagittal plane to reduce through plane motion. Eight imaging slices were acquired at each time point, with 2.5 × 2.5 × 4.68 mm^3^ voxels, reconstructed to 1.56 × 1.56 mm^2^ in-plane resolution and a slice thickness of 4.68 mm with 0 mm gap between them (time to echo (TE) = 75 ms, inversion time (TI) = 260 ms, repetition time (TR_min_) = 3000 ms, sensitivity encoding (SENSE) factor 2.3).

It was hypothesised that as the lymph nodes that receive lipids from the meal, they would physically swell, causing an increase in size. Three parameters (area, major axis, and minor axis) were recorded in an attempt to characterise any changes in lymph node size following the high fat meal. The number of lymph nodes visible at each time point was also compared in order to assess whether some lymph nodes which in the fasted state were not visible, then be-came visible, as they receive lipids.

It was also hypothesised that ADC may change as the lymph nodes receive lipid rich lymph from the intestine. Two diffusion weightings (b = 0 and 600 s/mm^2^) were used to measure ADC across the eight slices.

### 2.5. Gastric Volume (T2)

Gastric emptying and small bowel water were also measured to monitor the progression of lipids through the GI system. Gastric volumes were determined using a coronal Half-Fourier Acquisition Single-shot Turbo spin Echo (HASTE) sequence. Twenty eight slices were acquired at each time point with 1.4 × 1.7 × 5 mm^3^ voxels reconstructed to 1 × 1 mm^2^ in plane resolution with a slice thickness of 5 mm and slice gap of 1 mm (TE = 96 ms, TR = 1262, SENSE factor 2.0).

### 2.6. Small Bowel Water Content (SBWC)

SBWC was determined using a single shot, fast spin echo sequence (Rapid Acquisition with Relaxation Enhancement, RARE) as previously described [26]. Briefly, 20 coronal slices (7 mm slice thickness, no gaps in-between slices, 0.78 mm × 0.78 mm in-plane reconstructed resolution) were acquired in a single breath hold (TR = 1169 ms, TE = 400 ms, acquired resolution = 1.4 mm × 1.76 mm, SENSE factor 2).

### 2.7. Data Analysis

For each lymph node, a region of interest (ROI) was drawn around the periphery of the node on the two diffusion weighted images using Medical Image Processing, Analysis and Visualization software (Version 9.0.0, National Institutes of Health) [27]. Area, major, and minor axes were calculated. The signals from the two b value images were used to calculate ADC using the formula ADC = −1/b_600_ln(S(b_600_))/S(b_0_)). Where a lymph node could be consistently imaged across all time points, the % change in each parameter relative to baseline per lymph node was calculated. This allowed for changes in smaller lymph nodes to be equally represented.

The position of each lymph node in terms of vertebra level was also recorded in an attempt to assess which lymph nodes were being imaged [8].

The gastric volumes were calculated by drawing a region of interest around the bright content of the stomach in MIPAV on the T2 weighted dataset. The total volume was a sum of the volumes measured from each of the image slices. SBWC measurement was analysed with in-house software that was previously described and validated [28].

The reproducibility of data acquired from ROIs drawn in MIPAV was determined by drawing ROIs around the same randomly selected 15 nodes three times respectively (one node per time point per participant). All repeats were performed by the same observer.

All figures were generated using GraphPad Prism version 7.0d (San Diego, CA, USA).

## 3. Results

### 3.1. Lymph Node Imaging

**Figure 1:**
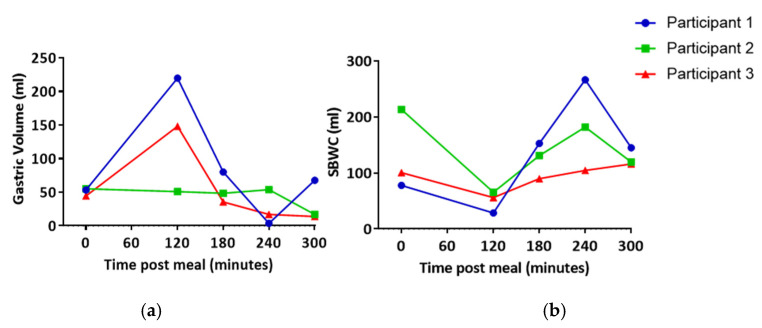
(**a**) Gastric volume and (**b**) small bowel water content (SBWC) post high fat meal. Each line represents data from an individual participant.

Gastric volume and SBWC were measured at several time points following the high fat meal in order to understand when lipids may be entering the intestinal lymphatics for each individual. In one participant, gastric volume was largely unchanged across all time points. However, in the other two participants, an increase in gastric volume was observed at 120 min post-meal (Figure 1). Gastric volume appeared to then be reduced to approximately baseline equivalent volumes at 180 min post-meal (Figure 1). Similarly, SBWC initially appeared to decrease in all subjects as a result of nutrient-driven fluid absorption [26]. This was followed by an increase at 180–120 min post-meal (Figure 1). Based on this, it was predicted that lipid uptake into the lymphatics would be the greatest after 120 min post-meal.

**Figure 2:**
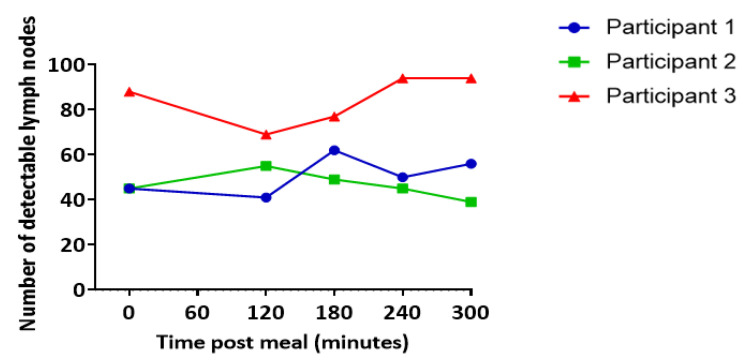
Number of lymph nodes visible at each scan. Data represent the combined total of nodes across all slices. Each line represents data from an individual participant.

The number of nodes visible in each scan ranged from 39 to 94 with an average of 61 nodes visible per time point per participant (mean ± SEM 4.27) (Figure 2). In two out of three participants, more lymph nodes were visible at 180, 240, and 300 min post-meal relative to the baseline (Figure 2).

The reproducibility of ROI drawing in MIPAV for 15 randomly selected nodes is demonstrated in Supplementary Table S1.

**Figure 3:**
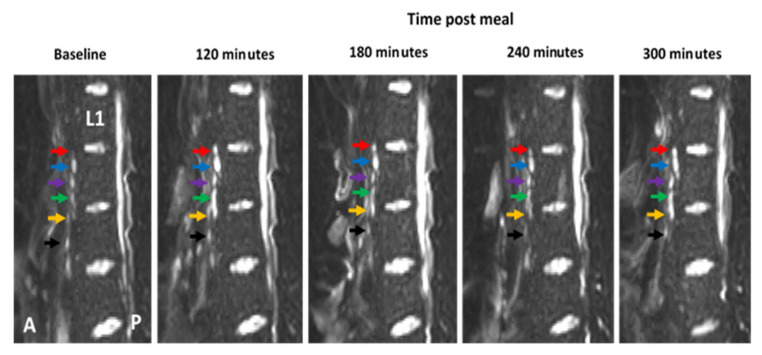
Example of diffusion weighted image (DWI) showing how the vertebra of the spine can be used as an anatomical marker to allow for the same lymph nodes to be consistently imaged at multiple time-points pre- and post-meal. The first lumbar vertebra is indicated (L1) and lymph nodes are indicated by coloured arrows. Colours represent the same individual lymph at each time point. The anterior side (A) of the body is to the left of each image and the posterior side (P) is on the right.

To control for any inconsistencies in the imaging planes at each time point, the vertebrae of the spine were used as an anatomical marker to ensure comparisons of “like for like” slices. The number of nodes consistently imaged across all time points was 11 for participant 1, 22 for participant 2, and 33 for participant 3, respectively (Figure 3). One node was excluded from the dataset based on fluctuations of over 200% change area at adjacent scanning time points, which was deemed physiologically impossible and likely to be the result of a partial volume error.

Lymph nodes that could be consistently imaged across all scanning time points were then analysed and compared at each time point relative to the baseline in an attempt to identify changes following intake of the high fat meal. All nodes were positioned anterior to the spine, with the majority being positioned between lumbar vertebrae 2 and 3 (Table 1).

### 3.2. Lymph Node Size

**Figure 4:**
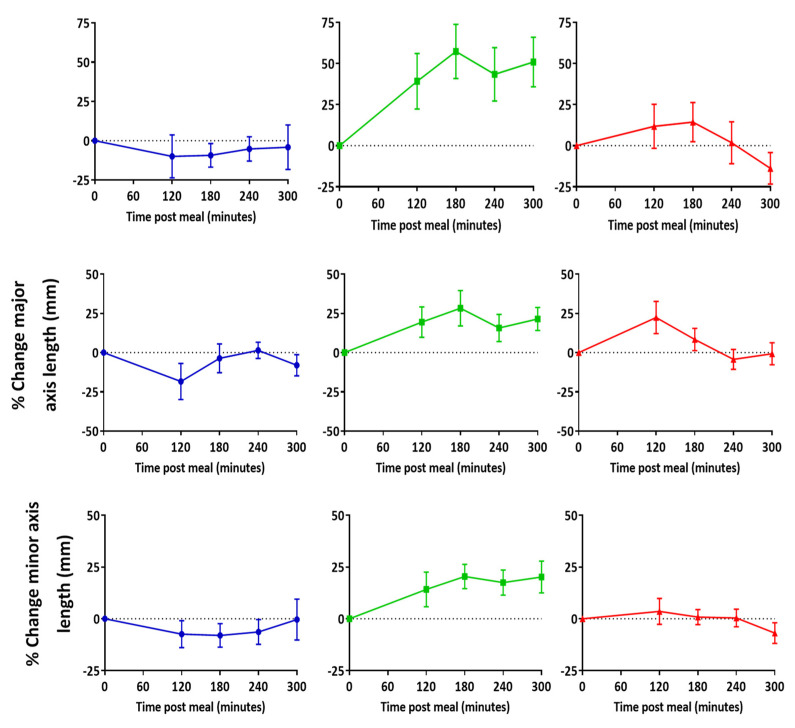
Change in lymph node area, major, and minor axes length relative to baseline post high fat meal. Data represent mean ± SEM of all nodes that were consistently imaged across all time points. *n* = 11 for participant 1, *n* = 22 for participant 2, and *n* = 33 for participant 3.

In two out of three participants, mean node area and major axes lengths increased at both 120 and 180 min post-meal relative to the baseline (Figure 4). However, minor axis length only increased above the baseline in one participant. There was no clear trend indicating a correlation between lymph node positioning relative to a vertebra level and change in size at each time point (Supplementary Figure S1). The largest increases relative to baseline were seen in the lymph nodes area, which was higher at both 120 min and 180 min post-meal in two out of three participants (Figure 4).

### 3.3. Apparent Diffusion Coefficient (ADC)

**Figure 5:**
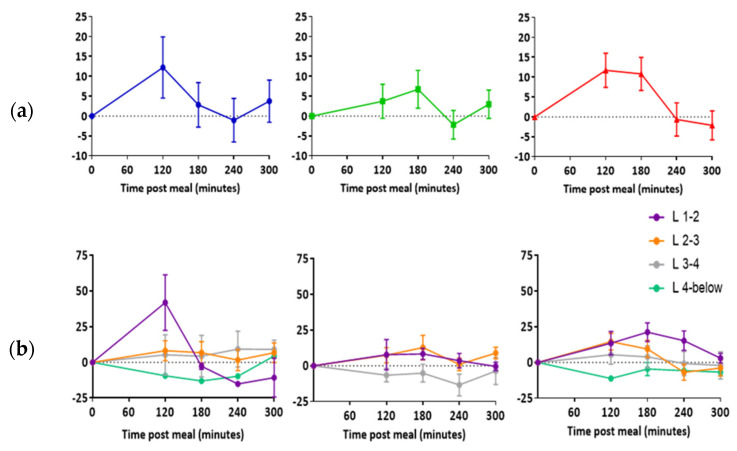
Change in lymph node apparent diffusion coefficient post high fat meal. (**a**) Data represent the average difference between individual lymph nodes (mean ± SEM). (**b**) Data represent the average difference between individual lymph nodes at each vertebra level (mean ± SEM). L = lumbar vertebra. *n* = 11 for participant 1, *n* = 22 for participant 2, and *n* = 33 for participant 3.

In all three participants, there was an increase in mean lymph node ADC post-meal relative to baseline (Figure 5a). Largest increases in ADC were observed in nodes located between the first and second and second and third lumbar vertebra (Figure 5b).

## 4. Discussion

The aim of this feasibility study was to establish whether MRI could be utilised to image the intestinal lymph nodes of healthy human volunteers at multiple time-points pre- and post-meal. Through repeat measurements of various parameters such as lymph node size and ADC, the feasibility of this method to identify changes in intestinal lymph nodes following the ingestion of a fatty meal was also assessed. Measurable changes in lymph nodes following lipid uptake could then be exploited to track the movement of lipids and drugs through the lymphatic system and inform which lymph nodes could be targeted using orally administered lipid-based formulations. To our knowledge, this work represents the first attempt to perform longitudinal imaging of the intestinal lymph nodes and measure lipid induced changes to lymph nodes in healthy human volunteers.

Data in the current study suggest that abdominal lymph node major and minor axes lengths may increase with the uptake of lipids. Larger changes relative to baseline were observed when comparing lymph node area than when comparing major and minor axes lengths. Area may therefore be a more sensitive measure for monitoring lipid uptake than axis length. This could be because both changes in major and minor axes lengths are simultaneously reflected in the area. ADC was the only parameter to consistently change across all participants. Increases in ADC were highest at 120 and 180 min post-meal, which encouragingly correlated with our time scale predictions on gastric volume and SBWC. Therefore, ADC may also be used to indicate lipid uptake into lymph nodes in future studies.

Using the MRI method described here, the vertebra of the spine could be used as an anatomical marker to allow for the repeated imaging of individual lymph nodes. The majority of the nodes imaged were positioned slightly anterior to the centre of the spine between the second and third lumbar vertebra. Based on their location, these nodes are most likely to be retroperitoneal nodes, more specifically the preaortic nodes. In some scans, nodes were visible closer to the spine and therefore more likely to be the retroaortic nodes. In addition, a small number of nodes were located between the fourth and fifth vertebra and likely to be the iliac nodes. Further imaging of these nodes in future work may help determine whether they can be targeted using a lipid-based formulation approach [8,29].

Lymph node imaging is routinely performed in the clinic to identify abnormalities that may indicate metastatic involvement from a primary tumour [30,31,32]. More recently, monitoring lymph node enlargement and necrosis have also been implemented in the staging and prognosis of inflammatory diseases such as Crohn’s disease and ulcerative colitis [23]. Several different methods have been employed for imaging lymph nodes including ultrasound, positron emission tomography (PET), computed tomography (CT), and contrast agent enhanced MRI [30]. These methods rely on the ability to detect changes in size or morphology, or in the case of ^18^F-FDG PET, altered physiological function. A number of studies have demonstrated that PET/CT fusion is more sensitive and accurate for lymph node staging than conventional CT [30]. However, the requirements for altered physiological function as well as exposure of the individual to radiation make these methods inappropriate for longitudinal imaging lymph nodes in healthy volunteers. In addition, for the purposes of this study, the use of contrast-enhanced MRI was ruled out on the basis that it requires IV or subcutaneous administration and is therefore not optimal for imaging in healthy human volunteers. Based on this, the major benefit of the MRI method described here is its non-invasive, label-free approach. This makes MRI a key tool for the serial scanning of healthy volunteers.

A number of approaches could improve the method sensitivity for detecting lipid-induced changes in lymph nodes. First, in terms of study design, the preliminary nature of this work means a small cohort of participants were included. Having indicated the feasibility of this method for tracking lymphatic uptake of lipids, more data from a larger number of participants would provide statistical power in future studies and thus aid conclusions about which lymph nodes can be targeted using lipid-based formulations. There is also substantial variability in the data for all parameters measured and although an initial appraisal of this is one of the findings of this feasibility study, future work with larger sample sizes will also allow for a more detailed assessment of this. More specifically, inter-participant differences in gastric emptying times were observed. Based on this, differences in the rates of lipid uptake into the lymphatics are also likely and so more personalised scanning intervals may ensure that differences are not being missed. Similarly, combining scanning with simultaneous blood sampling to determine triglyceride content would also provide indications of lipid entry into systemic circulation and subsequent flow out of the lymphatic system. In addition, in an attempt to enhance lipid uptake, the lipid content of the meal or formulation could be adjusted to include a higher lipid content. This may include ong-chain triglycerides, which have been shown in preclinical studies to enhance lymphatic uptake [33]. This, however, would require additional gastric emptying time data, which would likely be delayed as a result and may pose problems with palatability.

Improvements in imaging quality could also enhance the sensitivity of the proposed MRI imaging approach. Inevitably, as with most imaging techniques, a compromise between resolution and background noise is required. Although more invasive for participants, the use of an intravenous administration of an anticholinergic agent such as butylscopolamine could be utilised to prevent movement from peristalsis, which is known to decrease image quality [34]. Finally, although the vertebra of the spine was useful in identifying individual lymph nodes across each scan, the use of external markers to ensure consistent imaging planes would also be beneficial in future studies. A control scanning set in the fasted state, following water only, or alternatively following a meal with equal calories but no lipids, could also be useful to determine background fluctuations in lymph node parameters in the absence of lipids.

## 5. Conclusions

The purpose of this study was to assess the feasibility of MRI for repeated imaging of the intestinal lymph nodes in healthy volunteers, pre- and post-high fat meal. Individual lymph nodes could be repeatedly imaged at multiple time points. These data also suggest that postprandial increases in lymph node size and ADC could be observed. Based on this, the MRI methodology described represents a safe and label-free approach that shows promise for tracking the movements of lipids through the intestinal lymphatic system and interaction with immune cells. The method may therefore be used to inform which lymph nodes can be targeted using orally delivered lipid-based formulations. Future work should focus on ensuring the reproducibility in a larger participant cohort as well as optimisation of the methodology.

## Figures and Tables

**Table 1:** Vertebral level of nodes imaged across all time points. Data represent the mean across all participants ± SEM.

	Vertebra L1–2	Vertebra L2–3	Vertebra L3–4	Vertebra L4 & Below
Number of nodes visible	5 ± 1	11 ± 3	4 ± 1	1 ± 1
As a percent of total number of nodes visible	24%	52%	18%	6%

## Data Availability

The data presented in this study are available on request from the corresponding author.

## Supplementary Materials

The following are available online at https://www.mdpi.com/article/10.3390/pharmaceutics13091343/s1, Table S1: Relative standard deviation (RSD) and relative error (RE) for 15 nodes (*n* = 3), Figure S1: Percent change in area, major, and minor axes lengths.
