# Supplementary material for: Assessing Lymphatic Uptake of Lipids Using Magnetic Resonance Imaging: A Feasibility Study in Healthy Human Volunteers with Potential Application for Tracking Lymph Node Delivery of Drugs and Formulation Excipients
Source: Pharmaceutics. 2021 Aug 27;13(9):1343. doi: 10.3390/pharmaceutics13091343 (PMC8470042; doi:10.3390/pharmaceutics13091343)
Supplement: Supplementary file 1 [file pharmaceutics-13-01343-s001.zip › pharmaceutics-1299019-supplementary.pdf]

pharmaceutics

Supplementary Materials: Assessing lymphatic Uptake of Lipids Using Magnetic Resonance Imaging: A Feasibility Study in Healthy Human Volunteers with Potential Application for Tracking Lymph Node Delivery of Drugs and Formulation Excipients

Adelaide Jewell, Hannah Williams, Caroline L. Hoad, Paul R. Gellert, Marianne B. Ashford, James Butler, Snow Stolnik, David Scurr, Michael J. Stocks, Luca Marciani, Penny A. Gowland and Pavel Gershkovich

Table S1. Relative standard deviation (RSD) and Relative Error (RE) for 15 nodes (*n* = 3).

	Area		Major axis		Minor axis		ADC	
Node	**RSD**	**RE**	**RSD**	**RE**	**RSD**	**RE**	**RSD**	**RE**
1	20.20	11.66	15.12	8.73	16.74	9.67	17.60	10.16
2	8.17	4.71	5.70	3.29	5.13	2.96	0.67	0.39
3	0.00	0.00	1.29	0.75	1.30	0.75	0.67	0.38
4	14.39	8.31	3.96	2.29	17.99	10.38	3.22	1.86
5	29.99	17.32	18.38	10.61	17.02	9.83	3.09	1.79
6	14.89	8.59	3.67	2.12	17.99	10.39	0.73	0.42
7	8.41	4.85	5.07	2.93	22.43	12.95	0.98	0.56
8	13.61	7.86	12.55	7.25	4.95	2.86	1.58	0.91
9	6.43	3.71	3.79	2.19	2.58	1.49	1.10	0.64
10	11.66	6.73	4.31	2.49	7.81	4.51	12.11	6.99
11	16.23	9.37	10.03	5.79	8.68	5.01	1.18	0.68
12	44.04	25.43	35.89	20.72	8.41	4.85	6.57	3.79
13	9.94	5.74	10.05	5.80	3.89	2.24	5.18	2.99
14	25.12	14.50	10.77	6.22	19.27	11.12	4.19	2.42
15	12.86	7.42	8.61	4.97	13.90	8.02	18.96	10.94

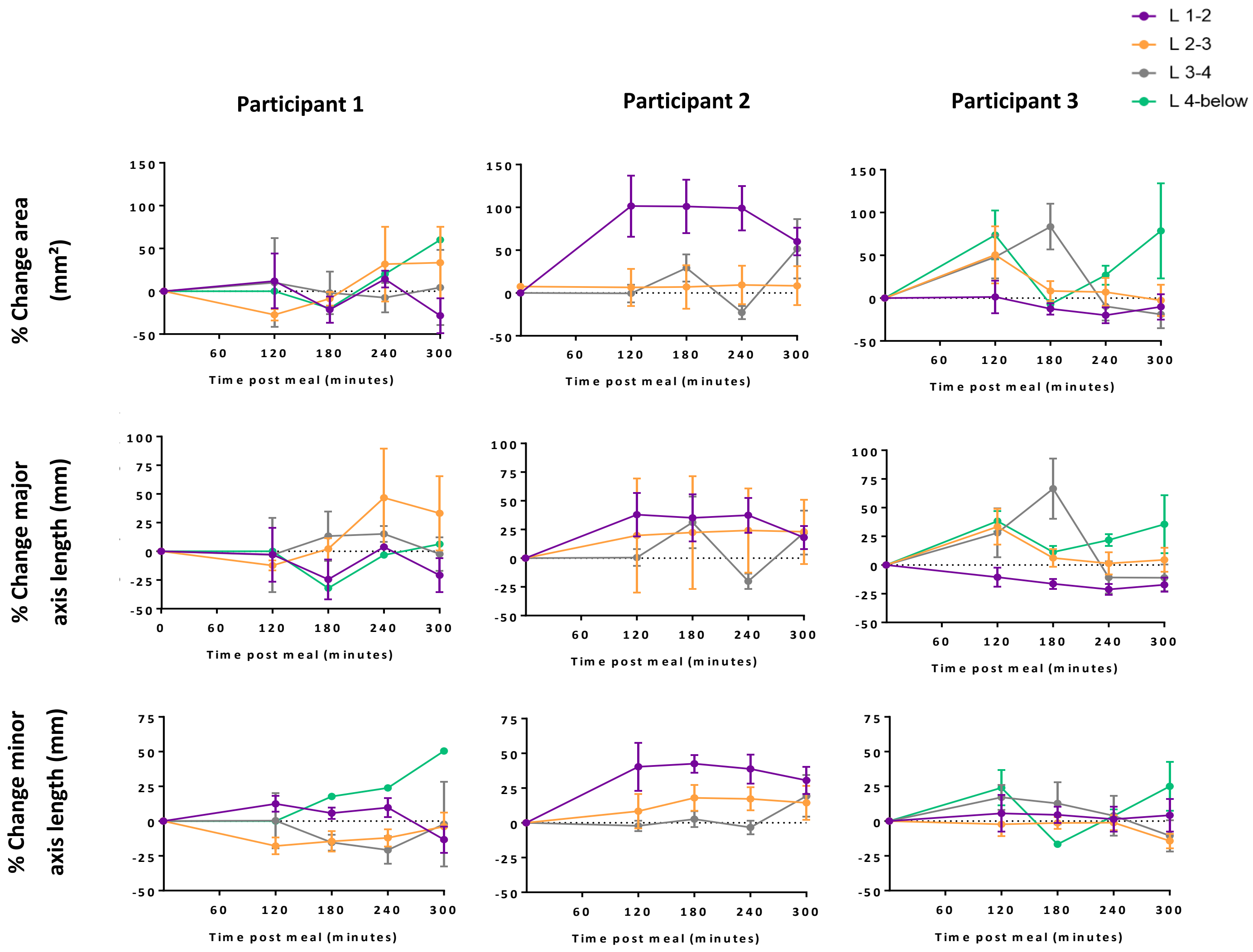

Figure S1. Percent change in area, major and minor axis length. Data represents the average difference between individual lymph nodes at each vertebra level (mean ± SEM). L = lumbar vertebra.
